# Acute Coronary Syndrome in Patients with SARS-CoV-2 Infection: Pathophysiology and Translational Perspectives

**DOI:** 10.37825/2239-9754.1034

**Published:** 2022-08-29

**Authors:** Francesco P. Cancro, Michele Bellino, Luca Esposito, Stefano Romei, Mario Centore, Debora D'Elia, Mario Cristiano, Angelantonio Maglio, Albino Carrizzo, Barbara Rasile, Carmine Alfano, Carmine Vecchione, Gennaro Galasso

**Affiliations:** a Department of Medicine, Surgery and Dentistry, University of Salerno, Baronissi, Salerno, Italy; b Vascular Pathophysiology Unit, IRCCS Neuromed, Pozzilli, Isernia, Italy

**Keywords:** Acute coronary syndromes, Myocardial infarction, Coronary artery disease, Novel coronavirus 2019, COVID-19, SARS-CoV-2, Endothelial dysfunction, Oxidative stress

## Abstract

Acute coronary syndromes (ACS) may complicate the clinical course of patients with Coronavirus Disease 2019 (COVID-19). It is still unclear whether this condition is a direct consequence of the primary disease. However, several mechanisms including direct cellular damage, endothelial dysfunction, in-situ thrombosis, systemic inflammatory response, and oxygen supply-demand imbalance have been described in patients with COVID-19. The onset of a prothrombotic state may also be facilitated by the endothelial dysfunction secondary to the systemic inflammatory response and to the direct viral cell damage. Moreover, dysfunctional endothelial cells may enhance vasospasm and platelet aggregation.

The combination of these factors promotes atherosclerotic plaque instability, thrombosis and, consequently, type 1 myocardial infarction.

Furthermore, severe hypoxia due to extensive pulmonary involvement, in association with other conditions described in COVID-19 such as sepsis, tachyarrhythmias, anemia, hypotension, and shock, may lead to mismatch between oxygen supply and demand, and cause type 2 myocardial infarction.

A deeper understanding of the potential pathophysiological mechanisms underlying ACS in patients with COVID-19 could help the therapeutic management of these very high-risk patients.

## 1. Introduction

After the detection of the first cases in Hubei Province, China, Coronavirus Disease 2019 (COVID-19) spread rapidly worldwide and reached pandemic proportions [1,2].

Although mainly involving the respiratory apparatus, COVID-19 can assume the characteristics of a multi-systemic disease involving many organs and lead to death in 15% of hospitalized patients [3]. Among these systemic effects, cardiovascular involvement is frequently reported and impact negatively on patient clinical outcome [4–13].

Although a clear association between these two conditions has not yet been found, a significant proportion of patients with COVID-19 may be complicated by acute coronary syndromes (ACS) [14,15], this aspect must be hardly considered, since cardiovascular risk factors are widely represented in COVID-19 patients. As reported in other infectious diseases, COVID-19 may promote atherosclerotic plaque instability resulting in rupture, thrombus formation and type 1 myocardial infarction (MI) [16,17]. However, in the context of a systemic disease, other factors may contribute to the ACS pathophysiology including cellular damage mediated by the virus or systemic inflammatory response, microvascular thrombosis, endothelial dysfunction and the mismatch between oxygen demand and availability secondary to respiratory involvement [18–22].

The aim of this review is to describe the potential pathophysiological pathways of ACS in COVID-19 patients, focusing on the translational application and on possible therapeutic perspectives.

## 2. Pathogenesis and transmission of COVID-19

COVID-19 is caused by severe acute respiratory syndrome coronavirus-2 (SARS-CoV-2) [23]. The internalization of the virus in the target host cells is mediated by the interaction between the angiotensin converting enzyme 2 (ACE2) receptor expressed by the cells, and the spike (S) protein expressed by the virus [24,25]. In this context, interferon release enhances the expression of further ACE2 receptors on the membranes of nearby pneumocytes and promotes the inflammatory response and the development of interstitial edema, microvascular thrombosis, and alveolar damage, which combined can precipitate the onset of an acute respiratory distress syndrome (ARDS) [26]. Viral transmission occurs through close individual contact and is mediated by respiratory droplets and subsequent inhalation of viral particles [27]. After an average incubation of approximately five days, the early clinical presentations of the disease are similar to other viral respiratory syndromes, including fever, cough, shortness of breath, fatigue, myalgias, headache, and gastrointestinal involvement [1]. The subsequent progression can be extremely wide, varying from asymptomatic or minimally symptomatic to life-threatening or fatal conditions, characterized by systemic inflammatory response syndrome, ARDS, multiple organ failure, and death [4,15,28].

## 3. The epidemiology paradox of ACS in COVID-19

Although ACS can complicate the clinical course of patients infected by SARS-CoV-2, a dramatic reduction in hospital admissions for ACS was observed during the first phase of the pandemic as demonstrated by the number of urgent or emergent coronary angiography performed [29–33]. Zhang et al. in a study involving 395 STEMI patients reported a halving of the number of primary percutaneous coronary interventions (pPCI) in 2020 compared to 2019 and 2018, and a concomitant increase in the use of fibrinolysis [34]. De Luca et al. also reported a significant reduction of pPCI performed in March and April 2020 compared with the same period of 2019 [35]. As expected, longer ischemia and door-to-balloon times were reported [36]. In contrast, fibrinolysis was frequently preferred as reperfusion strategy in patients with STEMI and COVID-19 [34,37–39].

This change in the rate of hospital admissions for ACS could have several explanations. Surely the governmental restrictive measures such as national lockdowns and the media emphasis on the rapid spread of the pandemic could have played a critical role in making the population more hesitant on seeking medical attention and into underestimating potentially life-threatening conditions. Moreover, the reorganization of health services and pathways has impacted on the efficiency of the emergency system, especially for time-dependent diseases such as STEMI [40]. This could also explain the significant increase in the incidence of out-of-hospital cardiac arrest, the most dreadful complication of ACS, which has been widely described in this period [35,41,42].

In addition, in the context of hospital access flows, it should be considered a substantial reduction in all cause access to the emergency cardiology department, which was more profound when COVID-19 cases increased and less evident during periods in which the epidemic curve tended to flatten [43].

## 4. Mechanisms of acute coronary syndromes in COVID-19

**Fig. 1:**
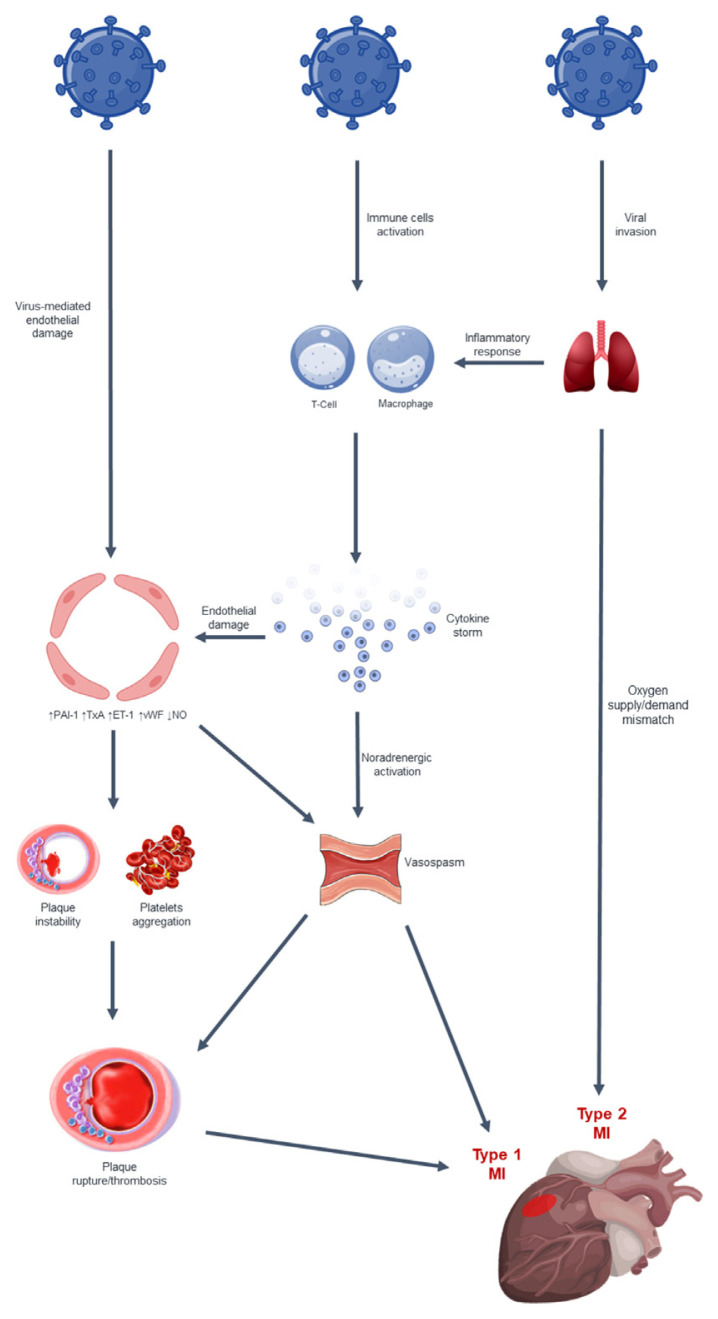
Pathophysiology of ACS in COVID-19. SARS-CoV-2, through ACE2 receptors, enters inside pneumocytes, macrophages, and endothelial cells. Hypoxia secondary to serious pulmonary involvement, which may escalate into ARDS, may cause type 2 MI due to oxygen supply-demand imbalance. Moreover, the excessive inflammatory response related to infection promotes the release of cytokines such as IL-1, IL-6, IL-7, TNFα, and IFNγ. The cytokine storm can facilitate the onset of endothelial dysfunction with subsequent production of oxidative and prothrombotic factors. Furthermore, the virus can directly damage the endothelium by interacting with its cells. Lastly, the state of hyperinflammation increases the activity of the sympathetic nervous system favoring coronary vasospasm. This context may then enhance atheromatous plaque rupture and platelet aggregation resulting in thrombosis and type 1 MI. ACS, acute coronary syndrome; ACE2, Angiotensin Converting Enzyme 2; COVID-19, Coronavirus Disease 2019; IFNγ, Interferon γ; IL-1, Interleukin 1; IL-6, Interleukin 6; IL-7, Interleukin 7; MI, myocardial infarction; SARS-CoV-2, Severe Acute Respiratory Syndrome Coronavirus 2; TNFα, Tumor Necrosis Factor α.

The characteristics of ACS in patients with COVID-19, such as angiographic evidence of un-obstructed coronary arteries, stent thrombosis, multiple thrombotic culprit lesions, and high thrombus burden, suggest distinct pathophysiological pathways [37,44,45]. (Fig. 1). MI may be also the first presentation of SARS-CoV-2 infection and may not correlate with the severity of lung involvement [44,46].

### 4.1. Hemostatic abnormalities

In hospitalized patients with COVID-19, multiple hemostatic abnormalities have been described and may be related to poorer clinical outcome. The most representative were decreased platelet counts, raised d-dimer serum levels, and prolonged prothrombin time [47,48]. Huang et al., in an observational study on hospitalized patients with COVID-19, reported that patients with higher levels of d-dimer were more likely to need intensive cares [49]. A study by Tang et al. also reported increased levels of d-dimer and fibrinogen degradation products, and a slight prolongation of the prothrombin time in patients hospitalized or who died due to severe forms of COVID-19 [50]. Whether these disorders are directly associated to the activity of the virus or are a consequence of an excessive inflammatory response secondary to the infection is yet to be defined [51]. By comparing laboratory blood samples obtained from patients with ARDS due to other causes and patients with ARDS due to SARS-CoV-2, patients with COVID-19 showed a significant increase in procoagulant and acute phase factors suggesting that the cytokine storm (CS) observed in these patients may act as a primary trigger in the development of thrombotic complications [52]. The systemic inflammatory response, through cytokines activity, may facilitate the expression of ultralarge von Willebrand factor multimers (ULVWF) and tissue factor (TF), which take part at various stages in the hemostatic mechanisms, enhancing thrombin production and the development of a procoagulant state [50,53–55]. A potential contributing factor to the development of SARS-CoV-2-related coagulopathy might be the presence of lupus anticoagulant (LA) [53], a condition that typically occurs in inflammatory and infectious disorders where cellular injury may expose phospholipid moieties, that are not usually reachable by the immune system, with consequent activation of the coagulation cascade and thrombus formation [56].

The presence of this procoagulant environment could explain the frequent occurrence of thrombotic complications such as venous thromboembolism (VTE), pulmonary embolism (PE), and ACS in patients with COVID-19 [7,18,22,44]. Furthermore, this patient population seem to have a peculiar phenotype in the development of ACS. Choudry et al., in a cohort of STEMI patients with COVID-19, reported an increased incidence of multiple thrombotic lesions, a higher thrombotic burden, and less successful PCI as assessed by the myocardial blush grade (a marker of myocardial perfusion) after pPCI, compared with a control group of SARS-CoV-2 negative STEMI patients [45,57,58]. In addition, Rodriguez-Leor et al. reported a high incidence of stent thrombosis (4.1%) during the hospitalization in patients with COVID-19 and STEMI [30], an event described in less than 1% in the general STEMI population up to 1 year from the index event [59–63].

### 4.2. Endothelial dysfunction

Vascular endothelium plays a crucial role in regulating the interaction between the circulatory system and tissues, and it is the main responsible for preserving vascular homeostasis by regulating vasomotility, immune response, platelet aggregation, coagulation, and vascular permeability. Endothelium can be harmed by several mechanisms, including oxidative stress due to an intracellular increase of superoxide anions, a condition already described in several conditions such as diabetes, hypertension, elderly age, and smoking. Hence, a dysfunctional endothelium will assume a predominantly vasoconstrictive and procoagulant state, promoting the development of ACS [64,65].

Endothelial dysfunction is also a key player in the pathophysiology of COVID-19 complications [66,67]. The endothelium can be impaired either directly by the virus or by the inflammatory response secondary to the infection, leading to venous, arterial, and microvascular thrombosis [21,68]. The endothelium increases the expression and release of TF, von Willebrand factor (vWf), thromboxane and plasminogen activator inhibitor-1 (PAI-1) [69–71] Furthermore, the CS favors the production of superoxide anions with consequent increase in oxidative stress and endothelial damage, creating a vicious loop that can lead to severe vascular complications [72–74]. An increased production and release of endothelin-1 also occurs in these circumstances, with consequent increased vasoconstriction and platelet aggregation [75].

These conditions could be exacerbated in patients with preexisting CV risk factors and CAD, favoring the precipitation toward an ACS or other thrombotic complications.

The role of the endothelium in the development of vascular complications encourages future investigations on endothelium-targeting therapy, including ACE inhibitors (ACEi) and statins [76–79].

### 4.3. Inflammatory response and cytokine storm

Inflammation is a leading mechanism in the development and progression of atherosclerosis [80]. During the acute phase of a viral infection, the CS can impair the physiological homeostasis by activating platelets, directly damaging the endothelium, and stimulating vasoconstriction by increasing sympathetic activity, facilitating the development of a pro-thrombotic state [16,81]. The interaction between these biological and mechanical agents can disrupt the atheromatous plaque architecture, facilitating the formation of thrombus and the occurrence of acute coronary syndrome [82,83].

An aberrant inflammatory response is typically associated with severe forms of COVID-19 [49,51]. Due to its self-expressing ability, interleukin-1 (IL-1) is the leading facilitator of CS, promoting the development of a self-enhancing inflammatory response [84]. The release of further inflammatory molecules, such as tumor necrosis factor (TNFα), interleukin-6 (IL-6), and various chemoattractant molecules, which facilitate tissue penetration of inflammatory cells is also promoted by IL-1 [85–87]. IL-6 further promotes the production of acute phase reactants, such as fibrinogen and plasminogen activator inhibitor-1 (PAI-1) priming a prothrombotic and antifibrinolytic state. Many studies have consistently reported increased levels of proinflammatory factors, such as IL-1, IL-6, IL-10, IFNγ, granulocyte colony-stimulating factor (GCSF), monocyte chemoattractant protein (MCP1), macrophage inflammatory protein 1 alpha (MIP1A), platelet-derived growth factor (PDGF), TNFα, and vascular endothelial growth factor (VEGF) in patients with COVID-19, suggesting the possibility of SARS-CoV-2-induced CS [49,88]. Cytokines impair the endothelial function and contribute to the development of thrombotic complications [50,53,89]. This evidence emphasizes the potential usefulness of an individualized therapy targeting these factors [90].

### 4.4. Oxygen supply/demand imbalance

Approximately 60% of patients with COVID-19 die due to hypoxemic respiratory failure [91]. The hypoxic state, in association with other disorders that may occur in critical COVID-19 patient, including sepsis, tachyarrhythmias, anemia, hypotension, and shock, may facilitate the onset of type 2 MI as a consequence of an imbalance between oxygen supply and demand [6,92]. The characteristics of these patients that are frequently elderly and with multiple comorbidities, justify the a poorer prognosis compared to patients with type 1 MI [93]. the comorbidities and frailty increase the risk of type 2 MI, a and contribute to the high mortality rate of these patients [4].

## 5. Myocardial infarction with non-obstructive coronary arteries

Non-obstructed coronary arteries have been widely reported in COVID-19 patients suffering an ACS, with a prevalence ranging from 30% to 40% [30,44,46,94] Different mechanisms may be responsible for the pathogenesis of myocardial infarction with non-obstructed coronary arteries (MINOCA), including plaque erosion, microthrombus formation, and coronary vasospasm [44,95,96]. In the context of COVID-19, the mechanisms of MINOCA are probably under-investigated due to difficulties of performing invasive and noninvasive tests that are time consuming and increase the exposure of the operator to the risk of contagion [97–102]. However, CS seems to be a key player also in MINOCA due to its potential to impair the endothelial integrity and function [103,104].

Takotsubo syndrome (TTS) is a particular condition that mimics ACS [105] clinical presentation and has been reported in 2–4% of hospitalized patients with COVID-19 [15,106–108]. This disease could also lead to severe complications and dramatically impact on the survival of these patients [109–113]. TTS could either be part of the pathophysiological manifestations of COVID-19 or related to the physical and emotional stress characterizing SARS-CoV-2 infection, leading to increased catecholamine levels [114]. Also, the psychological stress associated with COVID-19 spread may have contributed to the occurrence of TTS and justify the high incidence of TTS reported during the pandemic period [115,116].

## 6. Therapeutic perspectives

The need to limit the exposure of health workers to the risk of contagion has been a major limitation for the management of SARS-CoV-2 positive STEMI patients, particularly during the first peak of the pandemic. Scientific societies have suggested the use of fibrinolysis as primary treatment [117–119]; however, the increased risk of complications due to the delay to myocardial reperfusion does not seem to support this approach over pPCI, which has long been considered the standard of care [36,45,120,121].

On the other hand, in patients with non-STE and/ or equivocal ECG presentation, a rapid bedside echocardiogram showing regional wall motion abnormalities play an important role in detecting acute coronary syndromes early and triaging patients for invasive or conservative strategies [5].

Antiplatelet agents may have a particular value in the context of the thrombo-inflammatory syndrome frequently associated with COVID-19. Activated platelets may release several inflammatory mediators that may contribute to CS and [122], by interplaying with inflammatory cells, may further promote endothelial damage and subsequent thrombus formation [123,124]. Therefore, these therapeutic regimens could have pleiotropic effects in this particular STEMI population.

Anticoagulant drugs may also be particularly useful in COVID-19, since they may exert several anti-inflammatory effects. Unfractionated heparin (UFH) and low-molecular-weight heparin (LMWH) can affect the interaction between platelets and neutrophils and decrease the release of inflammatory mediators, such as IL-1β, IL-6, E-selectin, and ICAM-1 [125–127] In addition, heparins also possess direct antiviral effects, including interaction with heparan sulfate, a common component of many viruses essential for interactions with human host cells [128–130], and the ability to induce structural changes in the S1 subunit of SARS-CoV2, preventing virus access into the target cells [128].

However, it is still unclear what is the best anticoagulant molecule and its dosage [131,132]. A recent observational study of 4389 patients demonstrated that those receiving anticoagulant treatment had a lower mortality rate, with no difference between prophylactic and therapeutic dosing [133]. Randomized clinical trials are needed to define the best strategy for these patients, and the choice of antiplatelet/anticoagulant regimens should be individualized for every specific patient, taking into account his ischemic and hemorrhagic risk [134–137]. Table 1 summarizes the main ongoing trials testing antithrombotic and/or anticoagulant therapeutic regimens in patients with COVID-19.

Since endothelial dysfunction plays a prominent role in the development of ACS in the patient with COVID-19, ACEi and statins have been hypothesized to prevent the risk of ischemic complication [77,79].

The ACE2 receptor, used by SARS-CoV-2 to enter into the host cell, is not inhibited by ACEi and may have multiple beneficial effects [138]. These mechanisms are accomplished through several pathways leading to the production of the molecule Angiotensin 1–7 which exert multiple anti-inflammatory, antioxidant, vasodilator, and natriuretic effects [139]. SARS-CoV-2, by binding to ACE2 may interfere with these effects, causing its cleavage from the plasma membrane [140]. Conversely, ACEi may facilitate the overexpression of ACE2 by enhancing the transcription of its mRNA, leading to the extrinsicity of its beneficial effects [141]. Based on this evidence, the use of these medications might provide a viable preventive strategy in patients with COVID-19 at risk of developing ACS.

Statins are widely used in post–MI patients [142] and have shown to be also effective in regulating the inflammatory response at different stages of COVID-9 due to their ability to interfere with the Ras, Rho, and Rac GTPases and leading to a reduction in the expression of various transcription factors like NF-κB [143,144]. They may also enhance nitric oxide production by promoting the expression of eNOS with an overall antioxidant effect that restores normal endothelial homeostasis [145] At platelet level, statins were shown to reduce platelet reactivity and the releasing of proaggregative molecules like thromboxane, isoprostane and TF [146,147]. Similarly to ACEi, statins may also promote the expression of the ACE2 receptor [148]. Recent observational studies have reported that hospitalized patients with COVID-19 treated with statins showed a significantly lower mortality than patients not taking these medications [149,150]. However, their potential efficacy is still controversial [151] and randomized clinical trials are needed to clarify their effectiveness in these patients.

Beta-blockers (β-blockers) have been proposed in patients with COVID-19 to antagonize the hyperinflammatory response [152,153], since beta2-adrenergic receptors are expressed on several immune cells and their activation seems to promote inflammatory activity and cytokines release [154–157]. Therefore, targeting these receptors could be beneficial in counteracting the harmful effects of the inflammatory hyperactivation that occurs in these patients. However, their potential beneficial effect in patients with COVID-19 needs to be proven by randomized studies.

## 7. Conclusions

ACS may complicate the clinical course of COVID-19. These patients usually present a particular clinical phenotype compared to non-COVID-19 patients, with higher thrombotic burden, presence of multiple thrombotic lesions, poorer success of revascularization procedures, and higher incidence of non-obstructive CAD.

The pathophysiology of STEMI in SARS-CoV-2 positive patients is complex and includes hemostatic abnormalities, excessive inflammatory response, endothelial damage, and a mismatch between oxygen supply and demand. A deep knowledge of these mechanisms may be crucial for the management of these patients, and to plan a proper pharmacological treatment. Antiplatelet agents, anticoagulants, ACEIs, β-blockers, and statins may be helpful for preventing ACS in patients with severe forms of COVID-19, for reducing the risk of adverse events, and improving the patients’ clinical outcome.

However, further evidence from randomized studies is needed to substantiate their use in routine clinical practice.

## Figures and Tables

**Table 1:** Ongoing randomized clinical trials investigating antithrombotic regimens in patients with COVID-19.

Study name	Registration number	Population	Treatments	Design	Estimated enrollement (n)	Primary endopoint	Time of FU (days)
PARTISAN	NCT04445623	Non-ICU patients	Prasugrel 10 mg	Randomized, double blind	128	P/F ratio	7
PEAC	NCT04365309	Non-ICU patients	Aspirin 100 mg	Randomized, open label	128	Clinical recovery time The time of SARS-CoV2 overcasting	14 37
ACT-COVID19	NCT04324463	Non-ICU patients	Aspirin Rivaroxaban Colchicine	Randomized, open label, factorial	4000	Colchicine vs. control; Aspirin and Rivaroxaban vs. control: - composite of invasive mechanical ventilation or death - disease progression of 2 points on a 7-points scale Aspirin and Rivaroxaban vs. control: - composite of MACE (MI, stroke, acute limb ischemia, VTE, death).	45
C-19-ACS	NCT04333407	Non-ICU patients	Aspirin 75 mg Clopidogrel 75 mg Rivaroxaban 2.5 mg Atorvastatin 40 mg Omeprazole 20 mg	Randomized, open label	3170	All-cause mortality	30
RESIST	CTRI/2020/07/026791	Non-ICU patients	Aspirin 75 mg Atorvastatin 40 mg	Randomized, open label	800	Clinical deterioration expressed as progression of WHO clinical improvement ordinal score ≥6	10
COVID-PACT	NCT04409834	ICU patients	UFH iv Enoxaparin 1 mg/kg Clopidogrel 75 mg UFH sc Enoxaparin 40 mg/0.4 mL	Randomized, open label, factorial	750	Hierarchical composite: death due to venous or arterial thrombosis, pulmonary embolism, clinically evident DVT, type 1 MI, ischemic stroke, systemic embolism, or acute limb ischemia or clinically silent DVT	28

COVID-19, Coronavirus Disease 2019; DVT, Deep Vein Thrombosis; ECMO, Extra Corporeal Membrane Oxygenation; ICU, Intensive Care Unit; MACE, Major Adverse Cardiovascular Events; MI, Myocardial Infarction; P/F, PaO2/FiO2; RRT, Renal Replacement Therapy; SARS-CoV2, Severe Acute Respiratory Syndrome Coronavirus 2; UFH, Unfractionated Heparin; VTE, Venous Thromboembolism.
